# Morphometric Study of Sacral Hiatus in Dry Adult Human Sacra: Its Clinical Relevance in Caudal Epidural Block

**DOI:** 10.7759/cureus.29910

**Published:** 2022-10-04

**Authors:** Nisha Yadav, Vishal R Jasuja, Mamta Rani, Monika Srivastava, Nityanand Srivastava, Anurag Yadav

**Affiliations:** 1 Anatomy, Uttar Pradesh University of Medical Sciences, Etawah, IND; 2 Radiology, Uttar Pradesh University of Medical Sciences, Etawah, IND

**Keywords:** sacral hiatus variation, epidural anaesthesia, caudal block, sacral hiatus, sacrum

## Abstract

Introduction: Correct localization of the sacral hiatus is essential for administering a successful caudal epidural block. The present study was undertaken to find out the anatomical variations of sacral hiatus by a metrical method so that it could help anaesthesiologists in the clinical field.

Materials and methods: The study was performed on 140 (83 male and 57 female) adult human sacra. Various parameters of the sacrum studied were as follows: the shape of the hiatus, length of the sacral hiatus, transverse width at the base and anteroposterior diameter at the level of the apex. For each parameter, the mean value (calculated in mm), standard deviation, range and percentage of bones identified correctly were calculated.

Results: Various shapes of sacral hiatus were observed, including inverted “U” in 73 (52.14%), inverted “V” in 33 (23.57%), irregular in 10 (7.14%), elongated in 10 (7.14%) and dumbbell-shaped in 12 (8.57%). Absent sacral hiatus was observed in two (1.43%) specimens. The mean value for the length of sacral hiatus from the apex to the midpoint of the base was found to be 23.26 mm in males and 22.38 mm in females. However, the parameter was found to be statistically not significant. The mean value for transverse width at the base of hiatus was found to be 14.19 mm in males and 13.54 mm in females. The mean value for the anteroposterior diameter of the sacral canal at the apex was found to be 4.57 mm in males and 4.32 mm in females. Both the above parameters were found to be statistically not significant.

Summary and conclusion: The anatomical knowledge of sacral hiatus and its variations are important in caudal epidural anaesthesia, and it may improve the success rate of caudal epidural anaesthesia.

## Introduction

The sacrum is a large triangular bone forming the postero-superior wall of the pelvic cavity, wedged between the two innominate bones. It is formed by the fusion of five sacral vertebrae and forms the caudal end of the vertebral column. It has a base, apex, dorsal, pelvic, and lateral surfaces, and a sacral canal. On the dorsal surface below the fourth or third tubercle, there is an arched sacral hiatus in the posterior wall of the sacral canal, which is due to the failure of the laminae of the fifth sacral vertebra to meet in the median plane exposing the dorsal surface of sacrum [1].

The “sacral hiatus” is a commonly studied parameter of the sacrum. It is of immense value to anaesthesiologists and surgeons as caudal anaesthesia is administered through this route. Knowledge of the distance between the sacral hiatus and the dural sac is of clinical importance so that inadvertent iatrogenic injury to dural sac can be prevented during the procedure of caudal epidural block [2]. The present study was undertaken to find out the anatomical variations of sacral hiatus by a metrical method so that it could help anaesthesiologists in the clinical field.

This article was previously posted to the ResearchSquare preprint server on August 4, 2022.

## Materials and methods

Morphometric Study of Sacral hiatus of Human Sacrum, an analytical type of observational study was performed in the Department of Anatomy, on 140 (83 males and 57 females) adult human sacra. All the bones were dry, free from deformity and fully ossified. Clearance from the institutional ethics committee was obtained. Various parameters of the sacrum studied were as follows.

Shape

The shape of the hiatus was observed grossly.

Dimensions

**Figure 1:**
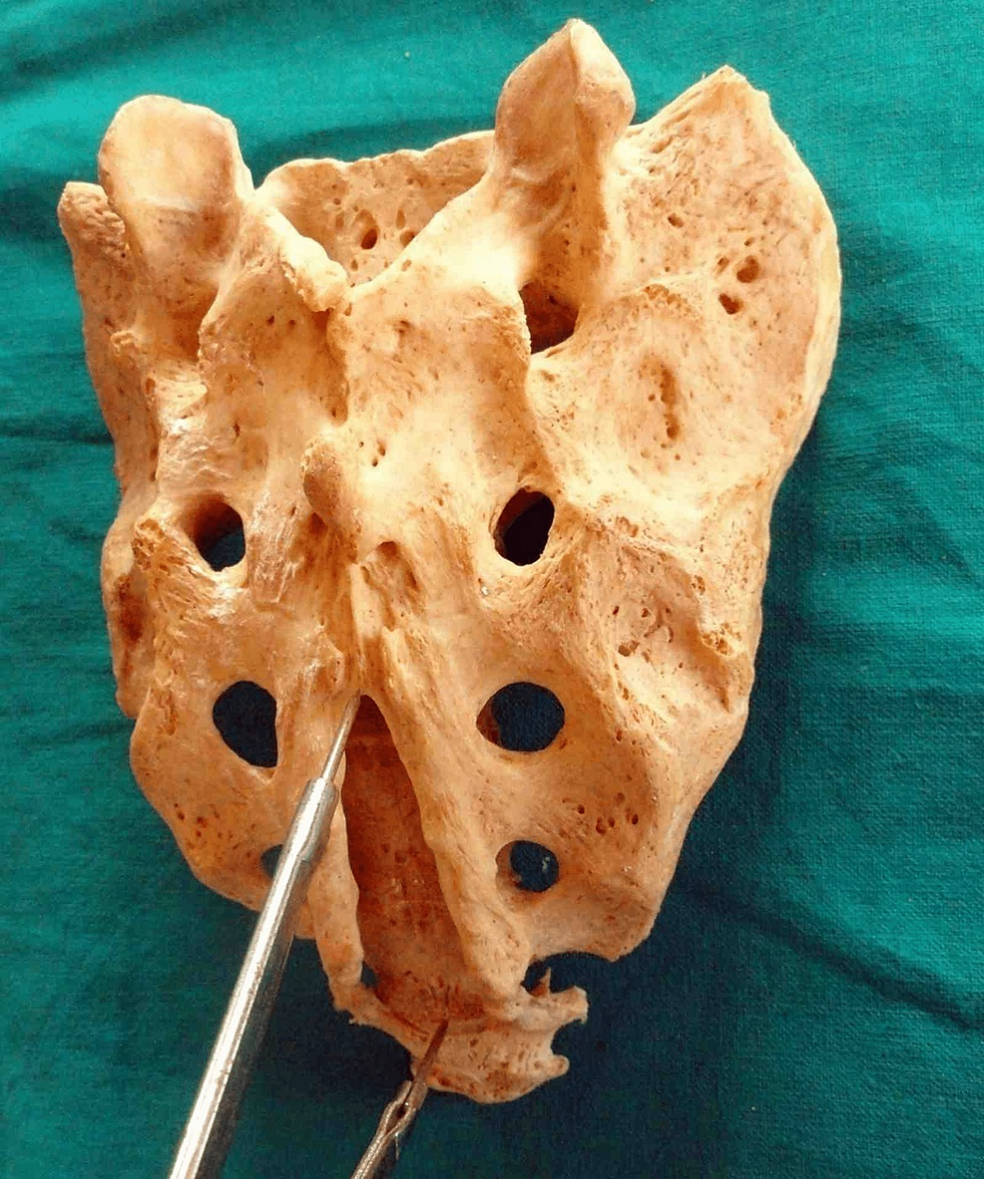
Measurement of length of sacral hiatus from apex to midpoint of base (elongated sacral hiatus)

Length is measured from apex to midpoint of base (Figure 1).

**Figure 2:**
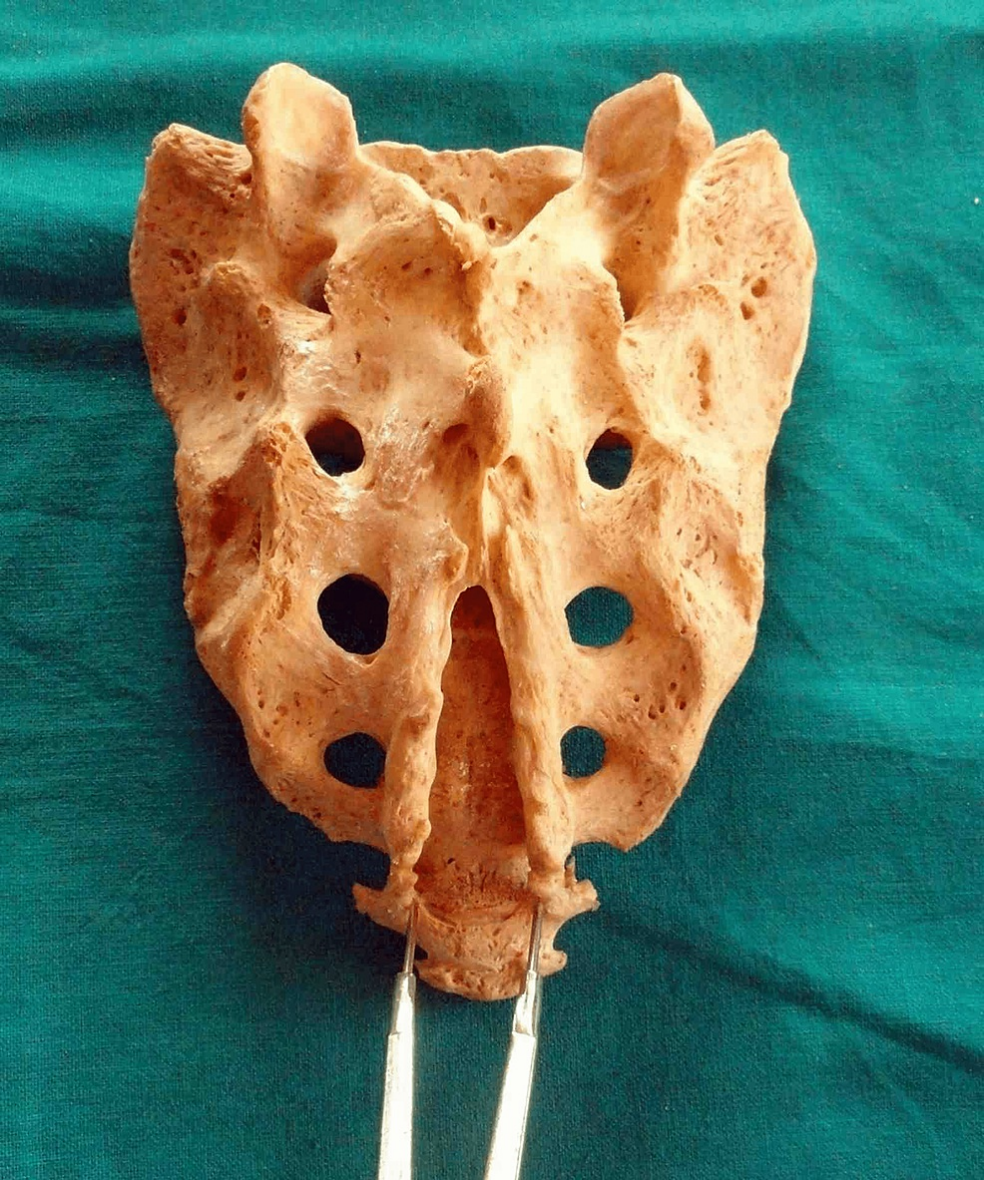
Measurement of transverse width at the base of sacral hiatus (elongated sacral hiatus)

Transverse width at the base is measured between inner aspects of the inferior limit of sacral cornua (Figure 2).

Anteroposterior diameter at the level of apex is recorded with a divider and steel measuring scale.

For each parameter, the mean value (calculated in mm), standard deviation, range and percentage of bones identified correctly were calculated. Statistical analysis was done by using unpaired t-test, to find out the differences, if any, in the mean values. All the calculations and statistical analyses were done using Microsoft Excel. A p-value of less than 0.05 (p < 0.05) was statistically significant, while p-value of less than 0.001 (p < 0.001) was statistically highly significant. Tables and graphs were generated using Microsoft Excel software.

## Results

**Figure 3:**
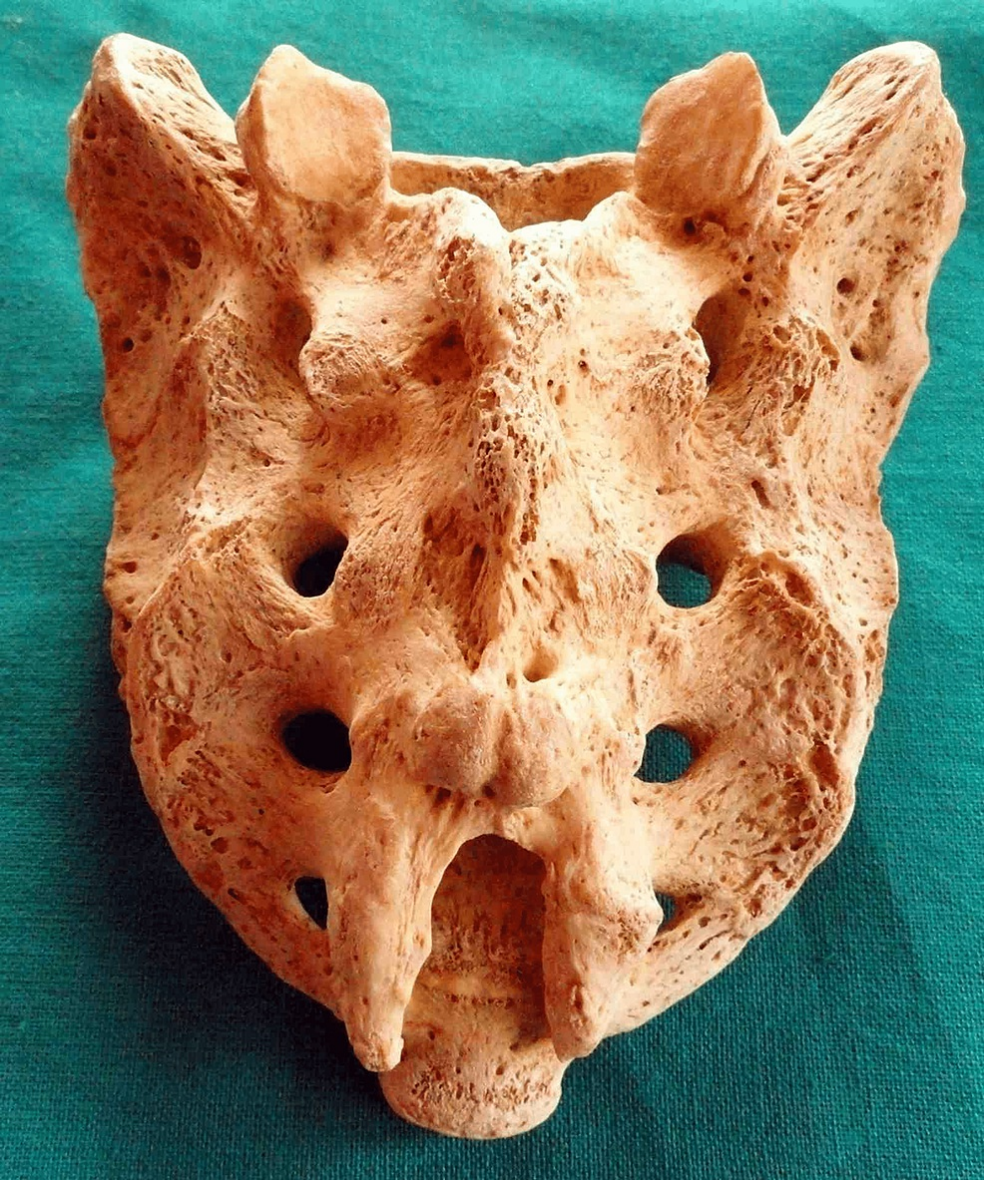
Inverted U-shaped sacral hiatus

The most common shape observed was inverted “U” in 73 (52.14%) of specimens (Figure 3).

**Figure 4:**
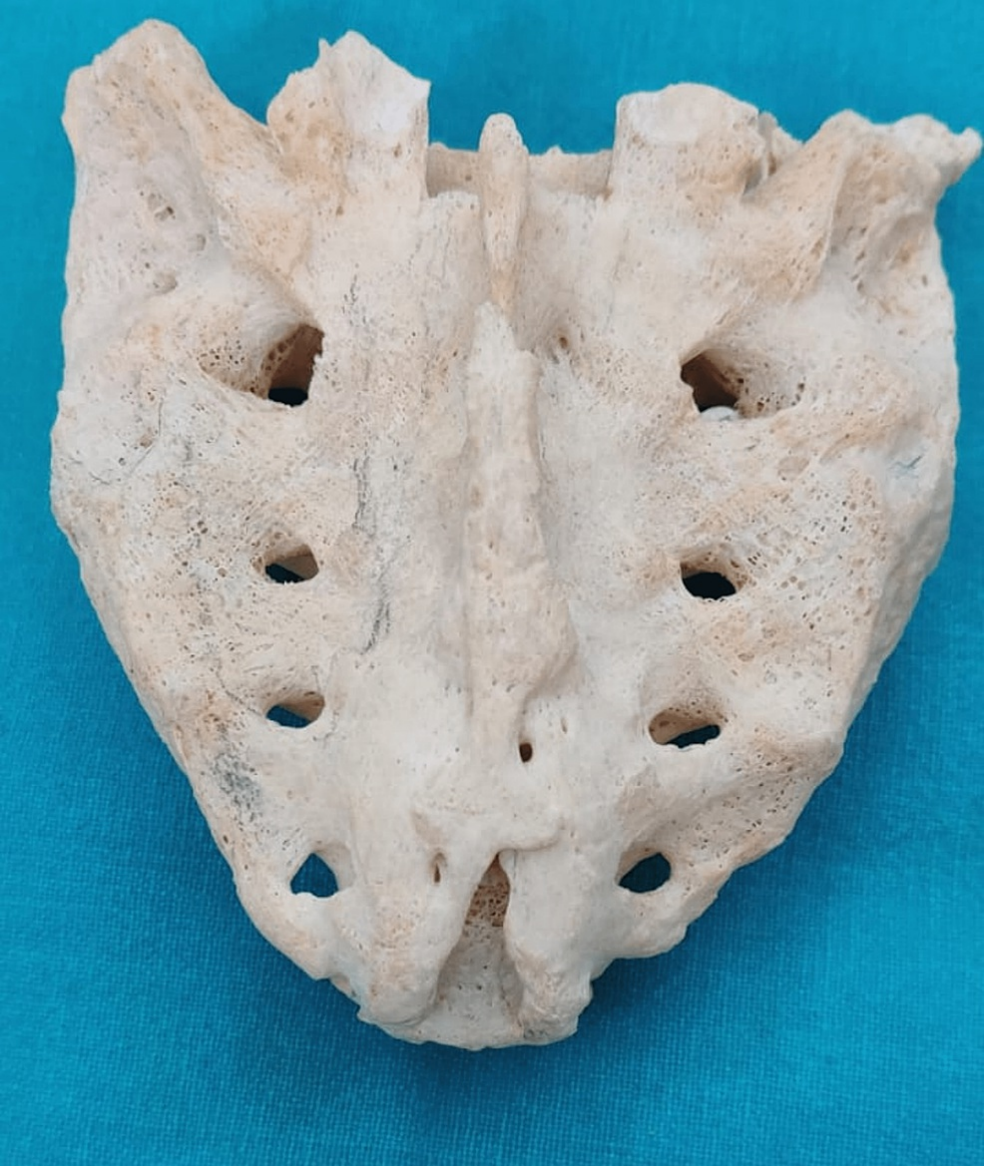
Inverted V-shaped sacral hiatus

The second most common shape observed was inverted “V” in 33 (23.57%) of specimens (Figure 4).

**Figure 5:**
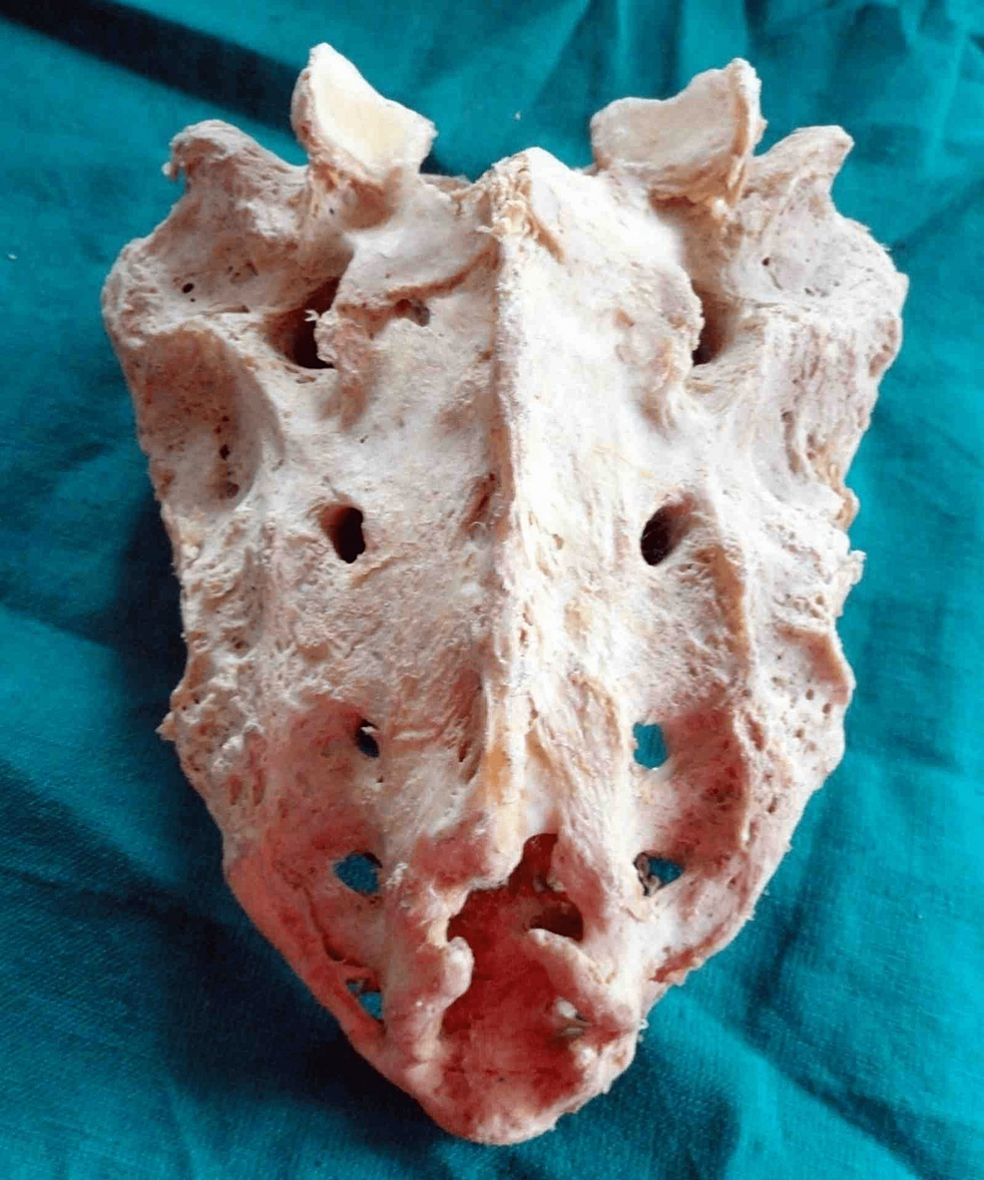
“Irregular” shaped sacral hiatus

Other shapes were also found during the observation. Figure 5 depicts irregularly shaped sacral hiatus.

**Figure 6:**
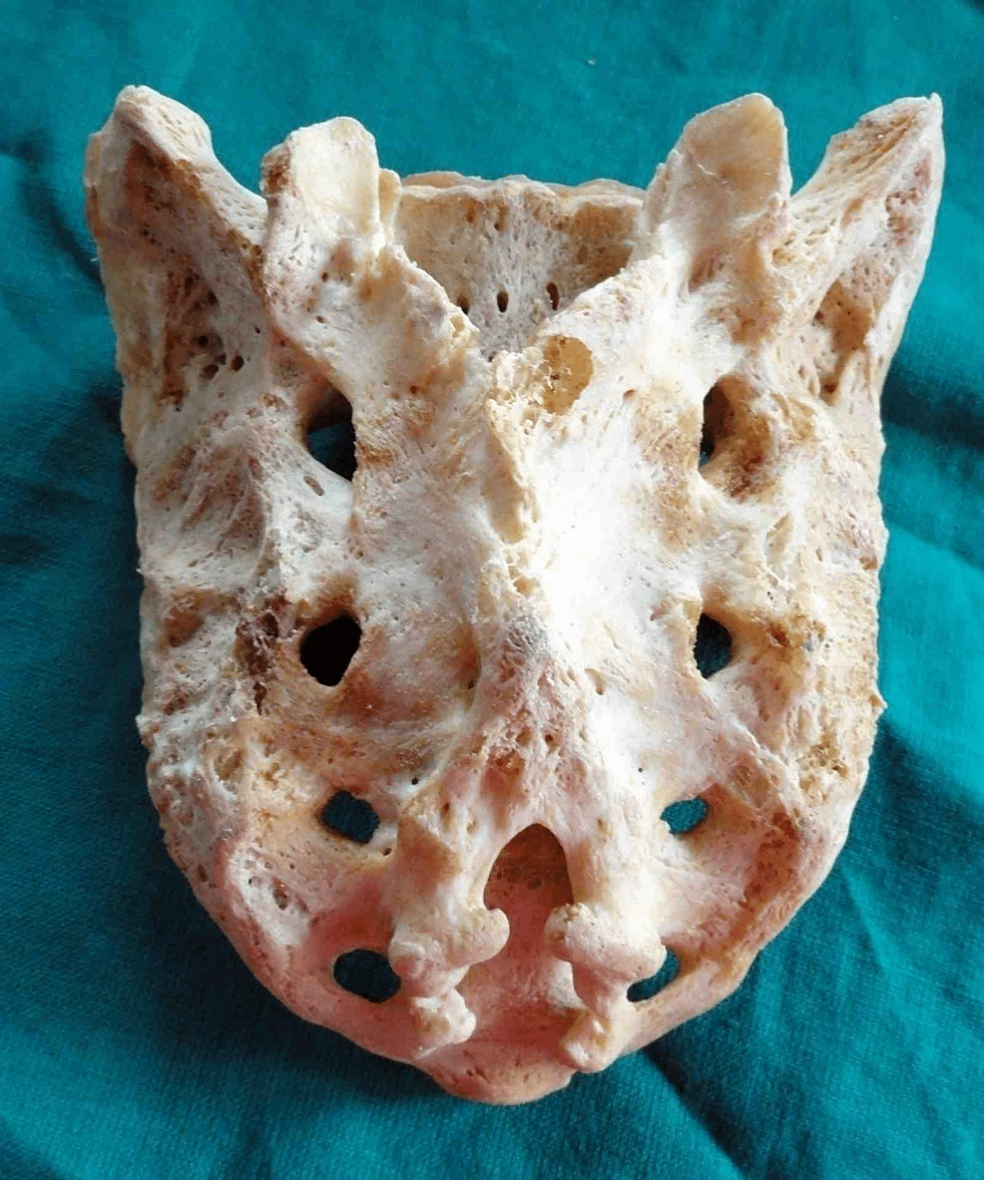
Dumbbell-shaped sacral hiatus

Dumbbell-shaped sacral hiatus is shown in Figure 6.

**Figure 7:**
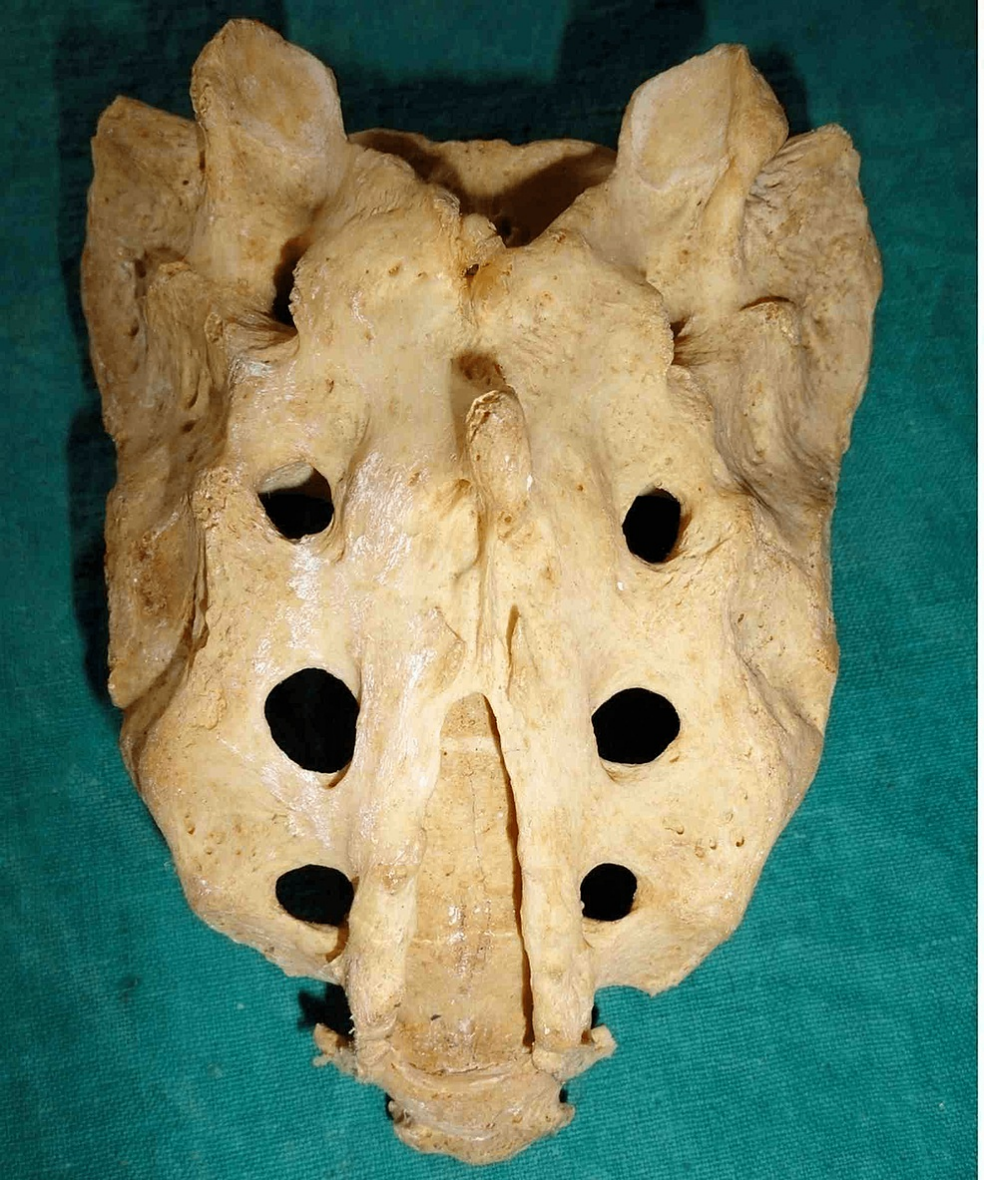
Elongated-shaped sacral hiatus

Elongated sacral hiatus is shown in Figure 7.

**Figure 8:**
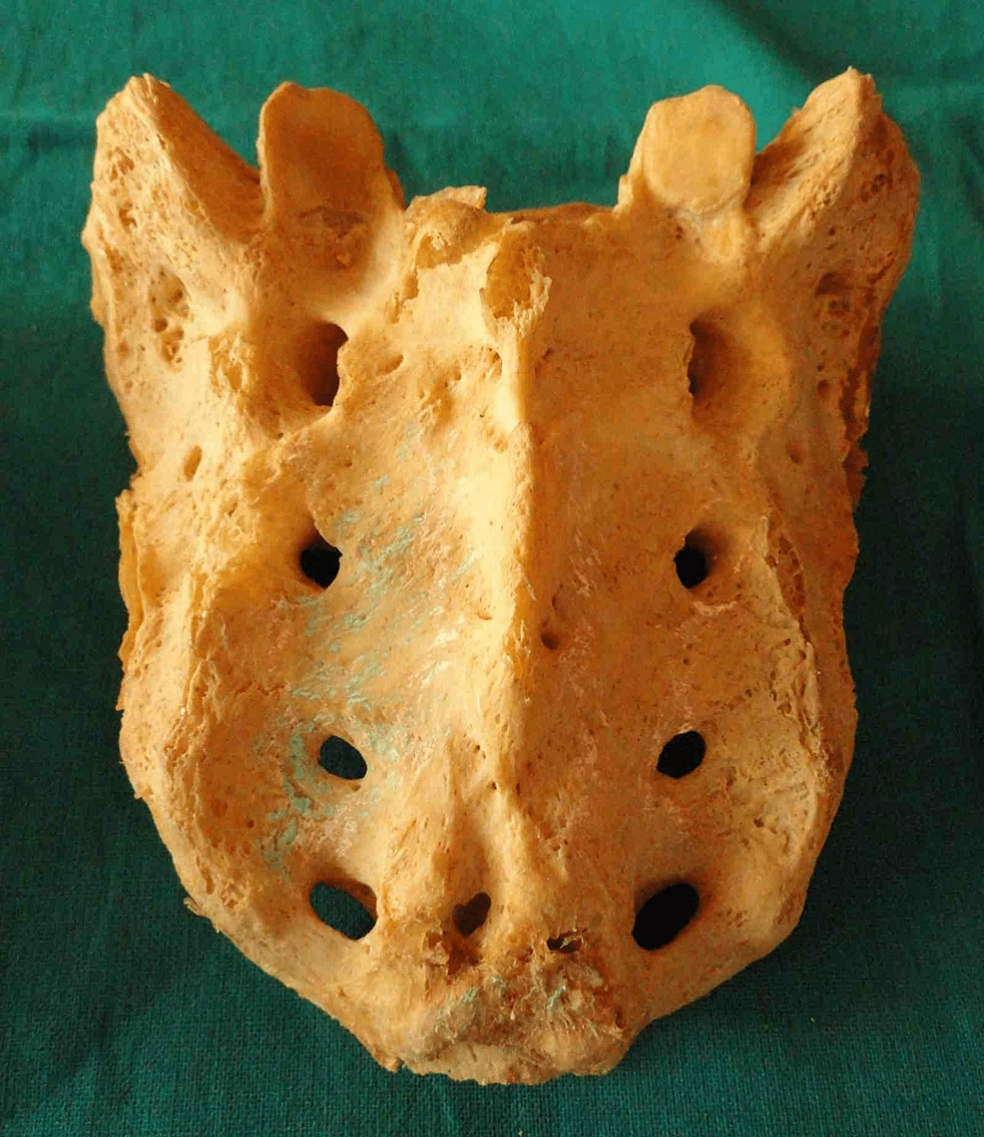
Absent sacral hiatus

Absent sacral hiatus was seen in two (1.42%) of cases (Figure 8).

Total 140 sacra were studied, and various shapes found are listed in Table 1.

**Table 1:** Shape of sacral hiatuses

Sr. no.	Shape	Number (140)	Percentage
1.	Inverted – U	73	52.14
2	Inverted – V	33	23.57
3.	Irregular	10	7.14
4.	Elongated	10	7.14
5.	Dumbbell	12	8.57
6.	Absent hiatus	2	1.43
	Total	140	100

In males, the length of sacral hiatus ranged from 6.99 mm to 51.08 mm with a mean of 23.26 ± 9.41 mm and in females it ranged from 6.58 to 41.03 mm with a mean of 22.38 ± 7.29 mm. On comparing the mean values in males and females, the measurement was found to be not significant, having a p value of 0.5546 (Table 2).

**Table 2:** Length of sacral hiatus from apex to midpoint of base * Not Significant

	Male	Female
Number of bones	82	56
Range	6.99-51.08	6.58-41.03
Mean	23.26	22.38
Standard deviation (S.D.)	9.41	7.29
t value	0.5924	
p value	0.5546*	

Transverse width at the base of sacral hiatus ranged from 4.61 mm to 19.11 mm, with a mean of 14.19 ± 2.61 mm in males and 5 mm to 19 mm with a mean of 13.54 ± 2.35 mm in females. On comparing the mean values in males and females the measurement was found to be not significant having a p value of 0.1340 (Table 3).

**Table 3:** Transverse width at the base of hiatus *Not Significant

	Male	Female
Number of bones	82	56
Range	4.61-19.11	5-19
Mean	14.19	13.54
Standard deviation (S.D.)	2.61	2.35
t value	1.507	
p value	0.1340*	

In males, the anteroposterior diameter of the sacral canal at the level of apex ranged from 2 mm to 8 mm with a mean of 4.57 ± 1.46 mm and in females, it ranged from 1 mm to 8 mm with a mean of 4.32 ± 1.62 mm. On comparing the mean values in males and females the measurement was found to be not significant, having a p value of 0.3438 (Table 4).

**Table 4:** Anteroposterior diameter of sacral canal at the level of apex * Not Significant

	Male	Female
Number of bones	82	56
Range	2- 8	1 – 8
Mean	4.57	4.32
Standard Deviation (S.D.)	1.46	1.62
t value	0.9500	
p value	0.3438 *	

## Discussion

The caudal epidural route was used for the first time for analgesia drug delivery into the epidural space through sacral hiatus in 1900 [3]. The technique of caudal epidural injection [4] was further developed in 1901. Continuous caudal epidural anaesthesia [5] was introduced later in 1942.

Epidural space is approached through sacral hiatus for giving analgesia and anaesthesia for various surgical procedures, treatment of lumbar spinal disorders and management of chronic back pain, administration of epidural anaesthesia in obstetrics for painless deliveries, perineal surgeries, colposcopy, orthopaedic procedures like treatment of sciatica to give corticosteroids injections, to provide pre and post-operative analgesia in adults and children [6].

A needle is passed through the skin, subcutaneous tissue and sacrococcygeal ligament and finally the caudal epidural space to achieve drug delivery into the epidural space [7]. When an ultrasound-guided caudal epidural block is performed, the success rate increases up to 100%, but it is not always possible to perform the procedure under the guidance of ultrasound, mostly due to the unavailability of an instrument [8].

Thorough and detailed knowledge of anatomical variant features of sacral hiatus will improve the success rate of caudal epidural block for anaesthesia and analgesia. According to Aggarwal et al. [9] depth of sacral hiatus, less than 3 mm may be the cause of failure of needle insertion. Surrounding bony irregularities, different shapes of sacral hiatuses and defects in the dorsal wall of the sacral canal should be taken into consideration before undertaking a caudal epidural block to avoid its failure. Mustafa MS et al [10] found that needle insertion into the sacral hiatus for a caudal block should be done at its base to avoid the anatomic variations of the apex.

In the present study, various parameters of sacral hiatus were studied and compared. Various shapes of sacral hiatus found are discussed in Table 5. Kumar et al. [11] in their study found an inverted “V” in 94 of the specimens studied, making it the most common of all the shapes of sacral hiatuses. Nagar et al. [12], Patel et al. [13], and Shewale et al. [14] also found inverted “U” as the most common shape followed by inverted “V” as the second most common one.

**Table 5:** The incidence of different shapes of sacral hiatuses as observed by various authors *n = number of bones studied

Sr. no.	Shape of sacral hiatus	Kumar et al. (1992) [11] , n* = 202		Nagar (2004) [12], n =270		Patel et al. (2011) [13], n=150		Shewale et al. (2013) [14], n=204		Present study, n= 140	
		No.	%	No.	%	No.	%	No.	%	No.	%
1.	Inverted- U	60	29.70	112	41.5	74	49.3	83	40.69	73	52.14
2.	Inverted- V	94	46.53	73	27	30	20	66	32.35	33	23.57
3.	Irregular	-	-	38	14.1	-	4	19	9.31	10	7.14
4.	Elongated	28	13.86	-	-	41	21.1	19	9.31	10	7.14
5.	Dumbbell	15	7.43	36	13.3	6	4	12	5.89	12	8.57
6.	Absent hiatus	2	0.99	-	0.7	2	1	2	0.98	2	1.42

Table 6 enlists a comparison of the length of sacral hiatus from the apex to the midpoint of the base as measured by different researchers. Kumar et al. [11] in their study found the mean value for the length of sacral hiatus to be 20 mm in males and 18.9 mm in females. Similarly, Shewale et al. [14] in their study found the mean value for the length of sacral hiatus to be 23.44 mm in males and 20.44 mm in females. Trotter and Lanier [15] found the mean value for length 24.8 mm in males and 19.8 mm in females.

**Table 6:** Comparison of length of sacral hiatus from apex to midpoint of base as measured by different researchers

Sr. no.		Name of the worker		Length from apex to midpoint of base(mm)			
				Mean		Range	
				Male	Female	Male	Female
1.		Trotter and Lanier (1945) [15]		24.8	19.8	-	-
2.		Kumar et al. (1992) [11]		20	18.9	3-37	9-36
3		Shewale et al. (2013) [14]		23.44	20.44	5-53.5	9-42
4.		Present study		23.26	22.38	6.99- 51.08	6.58-41.03

The comparison of transverse width at the base of hiatus is shown in Table 7. Kumar et al. [11] in their study found the mean value of transverse width at the base of hiatus to be 13 mm in males and 12.50 mm in females. Similarly, the study conducted by Shewale et al. [14] on 204 sacra showed the mean value of transverse width at the base of hiatus to be 13.68 mm in males and 13.45 mm in females. On comparing the findings of the present study with that of other studies, it was found that the mean value of the transverse width at the base of hiatus was more in males as compared to females.

**Table 7:** Comparison of transverse width at the base of hiatus

Sr. no.	Name of the worker		Transverse width at base of hiatus (mm)			
			Mean		Range	
			Male	Female	Male	Female
1.	Kumar et al. (1992) [11]		13	12.50	5-20	8-18
2.	Shewale et al. (2013) [14]		13.68	13.45	4.5-19	7.5-18
3.	Present study		14.19	13.54	4.61-19.11	5-19

Table 8 depicts a comparison of the antero-posterior diameter of the sacral canal at the level of the apex as observed by different researchers. Kumar et al. [11] observed the mean value of anteroposterior diameter at the apex to be 4.8 mm with a range of 0 to 12 mm. Similarly, Nagar [12] found the mean value of anteroposterior diameter at the apex to be 4.88 mm with a range of 2 to 14 mm. A glimpse at the table given below will reveal that the mean value of the anteroposterior diameter at the apex of the present study correlated quite well with those of other studies. Trotter and Letterman [16] found the mean value of anteroposterior diameter at the apex to be 5.3 mm with a range of 0 to 11 mm.

**Table 8:** Comparison of antero-posterior diameter of sacral canal at the level of apex

Sr. no.	Name of the worker		Anteroposterior diameter at apex (mm)	
			Mean	Range
1.	Trotter and Letterman (1944) [16]		5.3	0-11
2.	Kumar et al. (1992) [11]		4.8	0-12
3.	Nagar (2004) [12]		4.88	2-14
4.	Present study		4.57	2-8

The present study has some limitations as the study was done on a sample size of 140 adult sacra and did not include a sample from the pediatric population. Also, the bones studied belong to the Indian subcontinent and did not include other races. Thus, the results derived cannot be generalised for worldwide application.

## Conclusions

Various shapes of sacral hiatus were observed, including inverted “U” in 73 (52.14%), inverted “V” in 33 (23.57%), irregular in 10 (7.14%), elongated in 10 (7.14%) and dumbbell-shaped in 12 (8.57%). Absent sacral hiatus was observed in two (1.43%) specimens. Thus, it concludes that variations in the shapes of sacral hiatuses should be kept in mind for successful caudal epidural anaesthesia.
